# About Coin

**Published:** 1881-02

**Authors:** 


### ABOUT COIN.

"Is there any law or statute forbidding the melting or working up the coin of the United States?"

In reply to this question, Section 5459, Revised Statutes, says:

"Every person who frandulently, by any art, ways or means, defaces, mutilates, impairs, diminishes, falsifies, scales, or lightens the gold or silver coins which have been or may hereafter be coined at the mints of the United States, or any foreign gold or silver coins which are by law made current or are in actual use and circulation as money within the United States, shall be imprisoned not more than two years, and fined not more than two thousand dollars."

This, so far as I am aware, is the only law on this subject. It certainly does not forbid the melting of coin for the arts and manufactures.

